# Circadian regulation of osteoclast lysosomal–resorption machinery: implications for osteoporosis therapy

**DOI:** 10.3389/fcell.2026.1876518

**Published:** 2026-07-30

**Authors:** Chang Zhou, Yi Tang, Lijuan Zeng, Shuhui Xu, Yijing Xiang, Kangwen Ning, Shuang Zhang, Silu Peng, Sha Chen, Huiping Liu, Guomin Zhang

**Affiliations:** School of Integrated Chinese and Western Medicine, Hunan University of Chinese Medicine, Changsha, Hunan, China

**Keywords:** bone resorption, circadian clock, diurnal rhythm, lysosomes, osteoclasts

## Abstract

Osteoporosis (OP) is a systemic degenerative skeletal disorder characterized by reduced bone mass and compromised biomechanical properties, with its pathogenesis closely associated with excessive osteoclast activation and dysregulated bone resorption. Emerging evidence has revealed that the osteoclast lysosomal–resorption apparatus (LRA) serves not only as the principal effector system responsible for bone matrix degradation but also as a critical hub governing the circadian regulation of bone resorption. Disruption of circadian rhythms can impair LRA homeostasis and function, thereby promoting osteoclast hyperactivity and accelerating pathological bone loss. In this review, we systematically summarize the mechanistic roles of the LRA in osteoclastic bone resorption and comprehensively discuss the multilayered regulatory network through which the circadian clock modulates LRA activity. Particular emphasis is placed on the pathological significance of circadian clock–LRA interactions in distinct forms of osteoporosis. Furthermore, we highlight emerging therapeutic strategies targeting circadian regulation and lysosomal homeostasis restoration as potential approaches for osteoporosis intervention. Elucidating the mechanistic basis of the circadian clock–LRA axis will not only advance our understanding of osteoporosis pathogenesis and progression but also provide a theoretical framework for chronopharmacology and rhythm-based therapeutic interventions. These insights may ultimately facilitate the development of more precise and personalized strategies for osteoporosis prevention, treatment, and long-term management.

## Graphical Abstract

**Figure:**
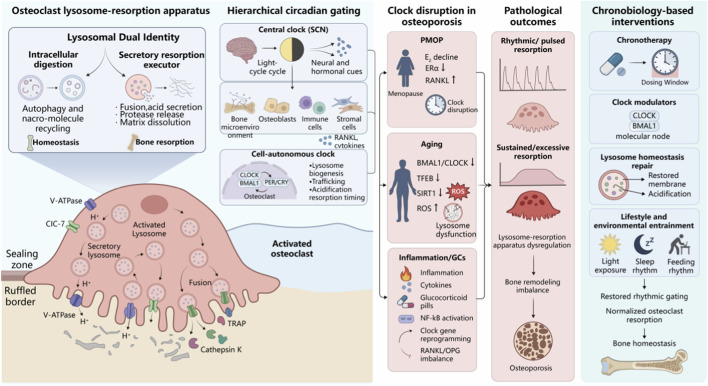

## 1 Introduction

Osteoporosis (OP) is a systemic degenerative bone disorder characterized by progressive bone loss and impaired biomechanical properties, leading to skeletal fragility and increased fracture risk (Salhotra et al., 2020; Yin et al., 2019). According to the diagnostic criteria established by the World Health Organization (WHO), osteoporosis (OP) affects approximately 19.7% [95% confidence interval (CI): 18.0%–21.4%] of the global population, with more than 200 million women currently living with the disease. Furthermore, among individuals aged over 50 years, nearly one-third of women and one-fifth of men are expected to experience an osteoporosis-related fragility fracture during their lifetime (Xiao et al., 2022). Given its high prevalence and devastating skeletal consequences, OP and the resulting fragility fractures have emerged as a major global public health challenge, imposing substantial and long-lasting burdens on healthcare systems, social welfare resources, and economic development worldwide (Xiao et al., 2022). Current clinical pharmacotherapies primarily include antiresorptive and anabolic agents. While their efficacy has been established, long-term or frequent use may be associated with adverse effects and compliance issues, highlighting the need to explore precise pathological mechanisms and actionable nodes underlying bone remodeling imbalance (Wang H. et al., 2023).

Osteoclasts (OCs) are the only multinucleated cells capable of efficiently resorbing mineralized bone and play a central role in skeletal growth and metabolism. The ruffled border of OCs represents a unique resorption apparatus (Ng et al., 2023). Within this structure, polarized transport and fusion of lysosome-related vesicles enable precise acidification and protease release in the resorption lacuna, making lysosomes the direct effectors of bone resorption (Jiang et al., 2024). The core functions of this lysosome–resorption apparatus (LRA) include: (i) establishing a localized acidic microenvironment via V-ATPase and other acidification systems to dissolve the inorganic hydroxyapatite phase, and (ii) directional exocytosis of lysosomes to release multiple hydrolases, such as cathepsin K (CTSK), which degrade the organic bone matrix, thereby completing the continuous “dissolution–cleavage–endocytic recycling” process (Jiang et al., 2024). Under physiological conditions, bone formation and resorption are balanced to maintain bone mineral density (BMD) and structural integrity. In postmenopausal, inflammatory, or metabolic pathological states, OC hyperactivation and enhanced LRA efficiency lead to sustained resorption pressure, driving the progression of OP (Xu et al., 2023).

The circadian clock is an endogenous timing system in mammals, comprising a hierarchical network of the central pacemaker located in the suprachiasmatic nucleus (SCN) and peripheral clocks distributed across tissues and organs. The SCN pacemaker consists of oscillatory neurons and astrocytes that sense and receive external light cues, synchronizing cell-autonomous clocks in peripheral tissues to align systemic rhythmic physiological activities with the light–dark cycle (Qin et al., 2023). Bone cells exhibit intrinsic circadian rhythmicity (Li T. et al., 2022). Both human OCs and osteoblasts display strong day–night activity patterns, peaking during the rest phase. OC resorption rhythms are mainly regulated by feeding cycles and fine-tuned by glucocorticoid levels, whereas osteoblast rhythms are controlled by glucocorticoids and sympathetic nervous activity (Winter et al., 2021). Since direct measurement of SCN activity in humans is not feasible, peripheral biomarkers such as melatonin and cortisol are typically used to infer circadian phase (Skubic et al., 2025). However, existing studies have yet to bridge the gap between biomarker rhythms and LRA execution mechanisms.

This review systematically summarizes the molecular mechanisms and circadian regulation of the OC LRA during bone resorption, highlighting the “dual identity” of lysosomes in OCs—performing conventional degradative functions while achieving precise bone matrix degradation through directional secretion. Key molecules, including Vacuolar-type H^+^-transporting adenosine triphosphatase (V-ATPase), chloride channel 7 (CLC-7), CTSK, and matrix metalloproteinases (MMPs), are discussed in the context of their coordinated roles in matrix degradation, emphasizing the centrality of acidification and protease output. Furthermore, the review details hierarchical regulation of the LRA by the SCN, peripheral, and cell-autonomous clocks and clarifies the pathological roles of circadian disruption in postmenopausal osteoporosis (PMOP), age-related osteoporosis (AROP), and inflammation- or glucocorticoid-associated osteoporosis. Finally, multidimensional strategies for OP intervention are proposed. Collectively, this review integrates structural and functional features of the OC LRA with circadian regulatory mechanisms, providing novel insights into OP pathogenesis and a theoretical basis for rhythm-targeted therapeutic approaches.

## 2 Lysosome–resorption apparatus in osteoclasts

### 2.1 Dual identity of lysosomes

In OCs, lysosomes possess a “dual identity.” The first identity is that of degradative lysosomes, representing conventional lysosomes, whose characteristic contents include acidic hydrolases such as cathepsin D (CTSD) (Zhao, 2012). These lysosomes play critical intracellular regulatory roles, participating in cellular metabolism, maintaining intracellular homeostasis (Ballabio and Bonifacino, 2020; Cao M. et al., 2021; Zhong and Richardson, 2025), and adapting to metabolic changes via membrane renewal (Lee and Overholtzer, 2021).

The second identity is that of secretory lysosomes, a specialized class of lysosome-related organelles (LROs) that OCs have evolved. These secretory lysosomes can form acidic vesicles that are directionally exocytosed at the ruffled border to release bone matrix–degrading enzymes, thereby playing a pivotal role in bone resorption (Van Meel et al., 2011; Xu and Teitelbaum, 2013; Baron et al., 1988). The primary distinction between degradative and secretory lysosomes lies in their content composition and abundance, with secretory lysosomes containing a richer proteome (Saftig and Klumperman, 2009).

**FIGURE 1:**
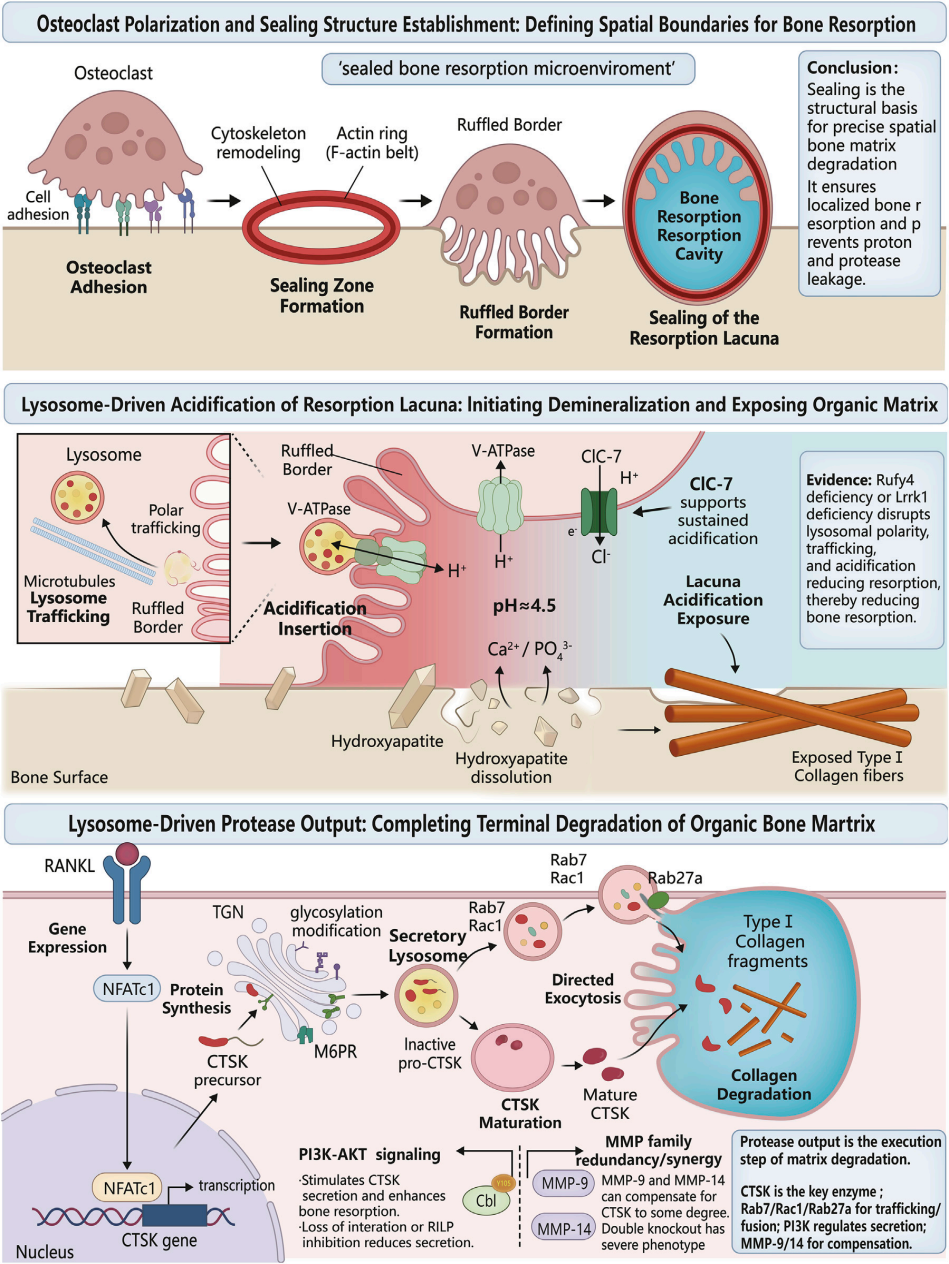
The process of bone resorption is mediated by the lysosomal–resorptive apparatus of osteoclasts. Sequential steps of osteoclast-mediated bone resorption. Osteoclasts establish a sealed resorption lacuna through polarization and sealing zone formation, followed by lysosome-dependent acidification to dissolve mineralized matrix and expose collagen fibers. Subsequent lysosomal exocytosis delivers proteases to degrade the organic matrix. Disruption of lysosomal trafficking, acidification, or protease secretion impairs bone resorption efficiency. Abbreviations: AKT, protein kinase B; ClC-7, chloride channel 7; CTSK, cathepsin K; F-actin, filamentous actin; LRRK1, leucine-rich repeat kinase 1; M6PR, mannose-6-phosphate receptor; MMP-9, matrix metalloproteinase 9; MMP-14, matrix metalloproteinase 14; NFATc1, nuclear factor of activated T cells 1; PI3K, phosphoinositide 3-kinase; Rab7, Ras-related protein Rab-7; Rab27a, Ras-related protein Rab-27A; Rac1, Ras-related C3 botulinum toxin substrate 1; RANKL, receptor activator of nuclear factor-κB ligand; RILP, Rab-interacting lysosomal protein; RUFY4, RUN and FYVE domain-containing protein 4; TGN, trans-Golgi network; V-ATPase, vacuolar-type H^+^-ATPase.

Stable lysosomal function is a prerequisite for OCs to perform normal bone resorption. Dysregulation of lysosomal activity directly disrupts the balance between osteogenesis and osteoclastogenesis, thereby impairing bone homeostasis and contributing to various metabolic bone disorders. For instance, overactive lysosomes in coordination with the resorption apparatus can lead to excessive bone matrix degradation and bone loss, ultimately resulting in osteoporosis. Therefore, elucidating the mechanisms of the OC lysosome–resorption apparatus and its role in bone resorption provides critical insights for novel strategies in OP prevention and treatment (Figure 1).

### 2.2 Key processes of lysosome-mediated bone matrix degradation

At the cellular level, the first step in OC–mediated bone matrix degradation is “sealing,” which involves forming a spatially confined compartment on the bone surface to allow the resorption apparatus to execute precise degradation. Upon adhesion to the bone, mature OCs assemble an actin-rich sealing ring around the ruffled border, together creating a relatively isolated resorption lacuna that defines the resorption boundary. In addition, the sealing ring acts as a barrier to prevent leakage of acidic molecules and hydrolases from the lacuna, ensuring efficient and localized bone resorption (Hou et al., 2023; Delaisse et al., 2021). This structural cooperation between the sealing ring and the ruffled border provides the foundation for spatially precise bone matrix degradation.

Next, lysosomes acidify the resorption lacuna via membrane ion transport, creating an optimal microenvironment for matrix degradation. Bone matrix consists of organic and inorganic components, mainly type I collagen (Col I) and poorly crystalline hydroxyapatite, respectively (Chen et al., 2025). Notably, degradation occurs in a defined sequence: upon lysosome fusion with the ruffled border, V-ATPase undergoes “transport–integration” and pumps protons into the lacuna. Concurrently, CLC-7, a lysosomal Cl^−^/H^+^ antiporter (Graves et al., 2008; Zifarelli, 2022), exchanges Cl^−^ and H^+^ at a 2:1 ratio across the lacuna membrane to maintain electrochemical neutrality (Tsukuba et al., 2017; Leisle et al., 2011), sustaining a strongly acidic environment (pH ≈ 4.5) that dissolves hydroxyapatite into Ca^2+^ and PO_4_^3-^ (Zhou N. et al., 2025; Salcedo-Betancourt and Moe, 2024). Only after mineral dissolution is Col I exposed (Delaisse et al., 2021) and subsequently degraded by lysosome-secreted CTSK (Mijanović et al., 2022). Thus, lysosomes not only generate the acidic milieu necessary for inorganic hydroxyapatite dissolution (Qin et al., 2025) but also maintain the activity of organic matrix–degrading enzymes, including cathepsins B, K, and L, which are particularly effective against Col I (Tsukuba et al., 2017). Disruption of lysosomal function or its membrane transporters inevitably impairs bone resorption. Supporting this, Minhee Kim et al. (Kim et al., 2024) demonstrated that deletion of RUN and FYVE domain-containing protein 4 (RUFY4), a key regulator of endosome–lysosome maturation, caused impaired lysosomal acidification, defective polarized trafficking, and reduced CTSK secretion, leading to markedly decreased OC resorptive activity in mice without altering OC number. Similarly, Sandi Shen et al. (Shen et al., 2023) found that leucine-rich repeat serine/threonine-protein (Lrrk1) knockout disrupted lysosomal localization and distribution, preventing V-ATPase enrichment at the ruffled border and severely inhibiting bone resorption, resulting in a high bone mass phenotype. These studies underscore the critical role of lysosome-mediated acidification in bone resorption.

Once Col I is exposed in the acidic lacuna, its degradation relies on lysosome-mediated “protease output,” the final and key step of matrix degradation. Two major lysosomal protein groups are involved: acidification proteins, exemplified by V-ATPase, and degradative proteases, including CTSK and MMPs (Jiang et al., 2024). CTSK, a lysosomal cysteine protease highly expressed in OCs, is a primary enzyme for Col I degradation (Dai et al., 2020; Tsukasaki et al., 2020). During late OC differentiation, Receptor activator of nuclear factor-κB ligand (RANKL) activates nuclear factor of activated T cells, cytoplasmic 1 (NFATc1), which binds to the CTSK promoter, initiating transcription and generating CTSK precursor protein (Costa et al., 2011). After glycosylation in the trans-Golgi network (TGN), the precursor is sorted via the mannose-6-phosphate receptor (M6PR) into secretory lysosomes (Ng et al., 2019; Ghosh et al., 2003). The proenzyme is activated in the acidic environment via autocatalytic cleavage to form mature CTSK (LaLonde et al., 1999), which is then directionally secreted into the sealed resorption lacuna through highly polarized exocytosis, coordinated by Ras-related protein Rab-7 (Rab7) and Ras-related C3 botulinum toxin substrate 1 (Rac1), with Ras-related protein Rab-27A (Rab27a) driving lysosome–membrane fusion (Jiang et al., 2024). CTSK subsequently performs spatially restricted degradation of the organic matrix within the acidic microenvironment. CTSK secretion and function are further regulated by lysosomal phosphoinositide 3-kinase (PI3K) activity (Costa et al., 2011). Similarly, inhibition of Rab-interacting lysosomal protein (RILP), an upstream regulator of PI3K–Akt signaling, decreases CTSK secretion (Wu et al., 2023), Considering the critical role of PI3K-Akt signaling in promoting CTSK release, this pathway may serve as a potential target for precise therapeutic modulation of osteoporosis through regulation of the LRA. Notably, Tang et al. (2025) provided translational support for this hypothesis by demonstrating that a cobalt–aluminum layered double hydroxide nanosheet (f-CA (OH)) selectively inhibited PI3K-Akt signaling and effectively restored trabecular bone architecture in ovariectomized mice. Collectively, these findings not only highlight the therapeutic feasibility of targeting PI3K-Akt signaling but also broaden the conceptual framework of LRA-oriented precision strategies for osteoporosis management.

In addition to CTSK, MMPs also contribute to organic matrix degradation and act synergistically. MMP family members specifically cleave the triple-helical domain of Col I (Yasuda et al., 2005). Even in CTSK deficiency, functional compensation within the MMP family, particularly MMP-9 and MMP-14, maintains bone remodeling capacity. Single knockout of MMP-9 or MMP-14 does not markedly alter bone mass, whereas double knockout significantly affects bone mass, highlighting functional redundancy (Zhu et al., 2020). Recent epigenetic studies by Zhu et al. (2022) further revealed that CTSK may function not only as a lysosomal protease but also as an epigenetic regulator, forming a cooperative network with MMP9 to modulate OC activation and matrix degradation.

However, bone resorption is an extremely energy-intensive process (Ledesma-Colunga et al., 2023). Therefore, the continuous execution of the aforementioned sealing–acidification–protein secretion cascade requires a sustained supply of adenosine triphosphate (ATP), for which mitochondria serve as the primary source. From the perspective of cellular energy metabolism, energy transfer and metabolic coupling mediated by the lysosome–mitochondria axis may represent a prerequisite for LRA-driven bone resorption. Recently, Qin et al. (2025) proposed the concept of the “lysosome–iron–mitochondria axis,” providing a novel framework for understanding the metabolic interplay between lysosomes and mitochondria. Their study suggested that iron acts as a central hub linking these two organelles. Interestingly, this concept complements the previously established “receptor activator of nuclear factor kappa-B ligand (RANKL)–mitochondria/heme axis” proposed by Qiu et al. (2025). As an essential substrate for heme biosynthesis, iron may serve as a key mediator through which lysosomal regulation of iron metabolism influences mitochondrial heme production. Based on these findings, a regulatory cascade involving the “lysosome–iron–mitochondria–heme” axis can be proposed. The proper functioning of this pathway may indirectly ensure the energy supply required for RANKL-induced osteoclast differentiation. In summary, lysosomes not only execute bone resorption through their acidification-dependent degradative functions but also support the high-energy demands of LRA activity by transmitting metabolic signals, such as iron, to mitochondria, thereby contributing to cellular energy homeostasis.

In summary, bone matrix degradation is a complex, multi-step, multi-molecular process rather than a simple absorption mechanism. Elucidating these molecular mechanisms is crucial for understanding bone resorption and developing precise, targeted interventions for bone mass modulation (Figure 1).

## 3 Hierarchical circadian regulation of the osteoclast lysosome–resorption apparatus

### 3.1 Central clock

The central circadian clock, located in the suprachiasmatic nucleus (SCN) of the hypothalamus, functions as the master circadian pacemaker of the body. By directly sensing environmental cues such as light exposure, temperature fluctuations, and feeding behaviors, the SCN synchronizes systemic circadian rhythms and coordinates temporal homeostasis throughout the organism (Qin et al., 2023). Accumulating evidence has established that bone metabolism exhibits pronounced circadian rhythmicity, particularly in bone resorption, which typically reaches its peak between 2:00 and 3:00 a.m. and displays a characteristic pattern of low daytime and high nighttime activity. In contrast, circadian oscillations in bone formation appear relatively modest (Tian and Ming, 2022; Darling et al., 2025). Given their central role in bone resorption, osteoclasts are widely regarded as the primary effector cells underlying skeletal circadian rhythms (Tian and Ming, 2022; Darling et al., 2025), whereas the LRA serves as the functional machinery responsible for the rhythmic execution of osteoclastic bone degradation. Nevertheless, direct mechanistic links between the SCN and the osteoclastic LRA remain largely unexplored, representing an important knowledge gap with substantial research potential. Future studies employing SCN lesion models and osteoclast-specific clock gene knockout mice, such as Bmal1^fl/fl^; Lyz2-Cre mice, are warranted to systematically elucidate the molecular mechanisms connecting central clock regulation with LRA rhythmicity. Such investigations would not only strengthen the molecular evidence supporting LRA-mediated circadian control of bone resorption but may also uncover novel therapeutic targets for osteoporosis. Despite the lack of direct evidence, the SCN, as the master circadian pacemaker, can indirectly communicate with peripheral skeletal clocks and cell-autonomous clocks through endocrine and sympathetic nervous system pathways (Astiz et al., 2019; Dibner et al., 2010; Oster et al., 2006; Moeller et al., 2022). These signaling networks may influence the rhythmic expression of LRA-associated genes and protein components, thereby orchestrating osteoclast rhythmicity at the systemic level.

Among these pathways, hormonal signaling represents one of the most important downstream outputs of the SCN. Glucocorticoids (Edmondson et al., 2026), parathyroid hormone (Fuleihan et al., 1997; Georg et al., 2024), and melatonin (Lu et al., 2025; Lu et al., 2020; Vasey et al., 2021) all exhibit robust circadian oscillations and are closely associated with bone metabolism, with glucocorticoids being particularly well characterized. As a hallmark endocrine output rhythmically released under the control of the hypothalamic–pituitary–adrenal (HPA) axis, glucocorticoids serve as critical mediators through which the SCN synchronizes peripheral circadian rhythms (Dibner et al., 2010; Oster et al., 2006). An increasing number of studies have demonstrated that glucocorticoids rhythmically regulate osteoclast function. Notably, the work of Fujihara et al. (2014) provided a relatively complete molecular framework linking glucocorticoids, clock genes, and the LRA. Following entry into osteoclasts, glucocorticoids initially activate the expression of clock genes. Subsequently, BMAL1 functions as a transcription factor that directly binds to the promoter region of CTSK, thereby driving its rhythmic transcription. More compellingly, adrenalectomy abolishes endogenous glucocorticoid production and disrupts Ctsk rhythmicity, whereas exogenous glucocorticoid administration restores this oscillation. These findings strongly suggest that glucocorticoids are indispensable for maintaining LRA rhythmicity. However, glucocorticoids also represent a well-established risk factor for osteoporosis. Clinically, prolonged glucocorticoid exposure markedly increases the incidence of osteoporosis and osteoporotic fractures (Hofbauer et al., 2025; Chen et al., 2023). Mechanistically, glucocorticoids exacerbate bone loss through dual actions: suppressing osteoblast proliferation and differentiation while simultaneously increasing the number and activity of mature osteoclasts (Hofbauer et al., 2025; Chen et al., 2023). Current evidence suggests that glucocorticoid-induced osteoporosis is largely mediated through upregulation of RANKL/osteoprotegerin (OPG) ratio (Piemontese et al., 2016; Cheng et al., 2022). Elevated RANKL subsequently binds to receptor activator of nuclear factor-κB (RANK) on osteoclasts (Li B. et al., 2022), activating the expression of key LRA-associated genes, including CTSK and ATP6v0d2, thereby enhancing osteoclastic bone resorption (Li et al., 2025; Gao et al., 2025). Collectively, the regulatory effects of glucocorticoids on LRA rhythmicity appear to be inherently bidirectional. Under physiological conditions, rhythmic glucocorticoid secretion is essential for maintaining normal circadian patterns of osteoclastic bone resorption. In contrast, under pathological conditions characterized by chronically elevated glucocorticoid levels or prolonged exogenous administration, glucocorticoid rhythmicity becomes disrupted. The resulting excessive pro-resorptive signaling, coupled with disturbed LRA oscillations, may accelerate skeletal deterioration and promote the progression of osteoporosis.

Parathyroid hormone (PTH) has likewise been demonstrated to exhibit a pronounced endogenous circadian rhythm, with circulating levels reaching their peak during the evening and nighttime periods (Fuleihan et al., 1997; Georg et al., 2024). This temporal pattern closely coincides with the onset of increased nocturnal bone resorption and is consistent with the well-established ability of persistently elevated, non-intermittent PTH to mobilize skeletal calcium and stimulate osteoclastic bone resorption (Bonnet et al., 2025). Similar to glucocorticoids, PTH promotes bone resorption, at least in part, through upregulation of the RANKL/OPG ratio (Chen T. et al., 2021). Beyond its canonical role in osteoclastogenesis, RANKL has been identified by Ferron et al. (2013) as an upstream regulator of transcription factor EB (TFEB), a master regulator of lysosomal biogenesis. Activation of the RANKL signaling pathway further enhances the expression of key LRA-associated genes, including Ctsk and ATP6v0d2 (Li et al., 2025; Gao et al., 2025). Therefore, PTH and other central hormones share some commonalities but are relatively independent. They can work together with various other factors to regulate the bone resorption rhythm of osteoclast LRA, making it exhibit a general pattern of being weak during the day and strong at night. Conversely, when PTH rhythmicity is disrupted and the bone microenvironment is chronically exposed to pathologically elevated PTH levels, excessive activation of the LRA may occur, substantially increasing the risk of skeletal fragility and fracture (Roumpou et al., 2025).

Importantly, the nocturnal peak of LRA-mediated bone resorption is not solely driven by pro-resorptive signals such as glucocorticoids and PTH. Rather, the maintenance of physiological bone resorption rhythms also depends on the counterbalancing anti-resorptive effects of melatonin. The characteristic circadian pattern of bone resorption, featuring lower daytime and higher nighttime activity, is therefore the result of dynamic temporal coupling and integrated actions among multiple regulatory factors. Under the control of the SCN, melatonin is a pineal gland-derived hormone characterized by a highly stable circadian secretion profile, with peak production occurring during the night (Lu et al., 2025; Lu et al., 2020; Vasey et al., 2021). Studies have shown that melatonin has a relatively obvious bone resorption inhibitory effect (Wu et al., 2024; Zhao et al., 2022), exhibiting a rhythmic bone protection effect related to LRA. Mechanistically, melatonin significantly suppresses the expression of ATP6v0d2, a critical subunit of the vacuolar H^+-ATPase (V-ATPase) complex responsible for osteoclastic acidification. By impairing acidification within the resorption lacuna, melatonin directly attenuates the bone-resorptive capacity of osteoclasts. Simultaneously, melatonin inhibits the mitogen-activated protein kinase/nuclear factor of activated T cells 1 (MAPK/NFATc1) signaling pathway, thereby further restricting the transcription of LRA-related genes at an upstream regulatory level (Kim et al., 2022). In addition, melatonin may regulate LRA rhythmicity through modulation of the clock gene BMAL1. Furthermore, an *in vitro* study by Wang X. et al. (2023) demonstrated that melatonin promotes apoptosis of RAW264.7 cells through the BMAL1/ROS/mitogen-activated protein kinase p38 (MAPK-p38) signaling pathway. Notably, BMAL1 is also a rhythmic regulator of CTSK, a key LRA-associated gene. Therefore, the effects of BMAL1 on bone resorption may be cell-stage dependent. During the osteoclast precursor stage, BMAL1-mediated signaling appears to primarily promote apoptosis (Wang X. et al., 2023), whereas in mature osteoclasts, BMAL1 may participate in the rhythmic regulation of LRA-associated molecules and bone-resorptive activity (Fujihara et al., 2014). The mechanistic basis underlying these stage-specific effects remains to be fully elucidated. Moreover, studies have suggested that appropriately timed melatonin supplementation may restore physiological or therapeutic nocturnal melatonin peaks, thereby exerting protective effects on skeletal homeostasis (Munmun and Witt-Enderby, 2021). Collectively, the rhythmic anti-resorptive actions of melatonin, in coordination with the LRA, are essential for maintaining the circadian homeostasis of bone resorption.

In addition to endocrine signaling, the central clock can transmit temporal information to skeletal tissues through the sympathetic nervous system. Previous studies have demonstrated that norepinephrine regulates RANKL expression via β2-adrenergic receptors and thereby influences osteoclastic bone resorption (Elefteriou, 2018). However, direct evidence linking sympathetic signaling to the osteoclastic LRA remains limited, and the underlying molecular mechanisms require further investigation.

**FIGURE 2:**
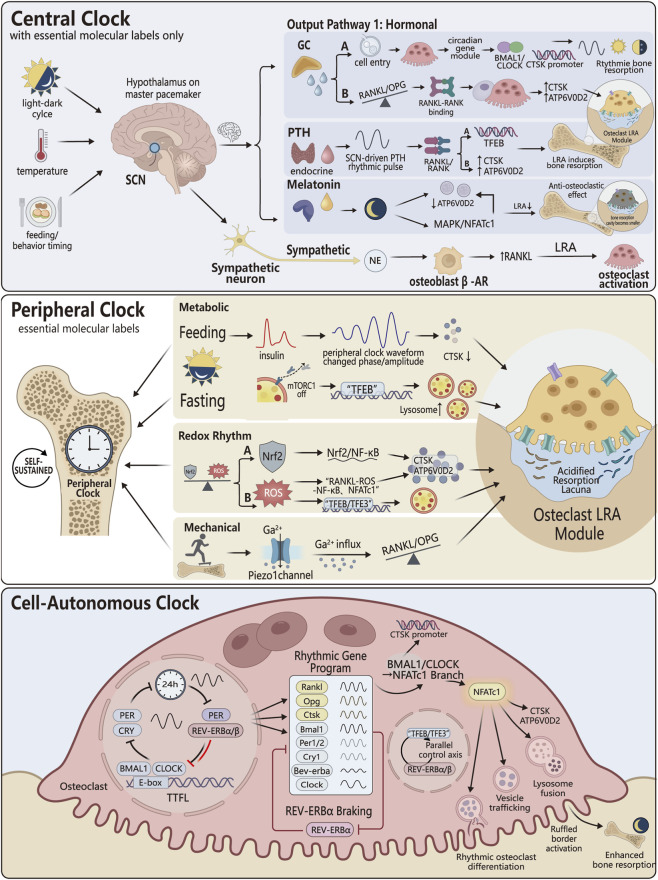
Circadian rhythms hierarchically gate and regulate osteoclast-mediated bone resorption. The circadian system regulates bone resorption via three tiers. The central clock (SCN) controls PTH, sympathetic, and hormonal outputs. Peripheral clocks integrate metabolic (insulin/fasting), redox (ROS/Nrf2), and mechanical (Piezo1) cues. The cell-autonomous clock drives rhythmic gene programs (BMAL1/CLOCK) and TFEB signaling for osteoclast differentiation. Abbreviations: LRA, lysosome-resorption apparatus; PTH, parathyroid hormone; SCN, suprachiasmatic nucleus; NE, norepinephrine; β-AR, beta-adrenergic receptor; ROS, reactive oxygen species; TFEB, transcription factor EB; GC, glucocorticoid; BMAL1, brain and muscle ARNT-like 1; CLOCK, circadian locomotor output cycles kaput.

Taken together, hormone-mediated signaling pathways represent the principal mechanism by which the SCN regulates the functional rhythmicity of the LRA. Whether through the rhythmic pro-resorptive actions of glucocorticoids and PTH or the nocturnal anti-resorptive effects of melatonin, temporal information originating from the central clock ultimately converges on key LRA components. This integration forms a dynamic “SCN–hormone–LRA” oscillatory axis through which bone-resorptive activity fluctuates in accordance with endocrine rhythms. Consequently, the LRA functions not only as the ultimate effector machinery of osteoclastic bone resorption but also as the terminal downstream platform through which the central circadian clock orchestrates skeletal resorption rhythms (Figure 2).

### 3.2 Peripheral clock

Peripheral clocks are widely distributed throughout diverse peripheral tissues and function as “24-h circadian relay stations” that bridge central clock signals with downstream cellular effectors. The skeletal peripheral clock possesses intrinsic oscillatory capacity and can be modulated by a variety of local and systemic cues, including metabolic and energetic changes induced by feeding or fasting, fluctuations in redox status within the local microenvironment, and mechanical loading imposed on the skeleton. These factors can alter both the amplitude and phase of skeletal peripheral clock oscillations, thereby influencing LRA activity and contributing to the rhythmic regulation of bone resorption.

In recent years, accumulating evidence has demonstrated that alterations in feeding schedules exert profound effects on metabolic health (Chawla et al., 2021; Reytor-González et al., 2025; Haupt et al., 2021), and can serve as potent zeitgebers for peripheral clocks (Pastore and Ballabio, 2019), regulating skeletal peripheral clock function through multiple mechanisms (Luo et al., 2021; Mühlbauer and Fleisch, 1995). An early study employing circulating bone turnover markers as outcome measures provided direct evidence linking feeding behavior to bone resorption. Bjarnason et al. (Bjarnason et al., 2002) identified a reproducible cyclical pattern, independent of age and sex, characterized by reduced bone resorption following food intake and restoration of resorptive activity during fasting. Notably, both endogenous and exogenous insulin stimulation partially reproduced this anti-resorptive effect, suggesting that insulin may serve as a critical mediator linking nutritional status to the circadian regulation of bone metabolism. The underlying molecular basis of this phenomenon was further elucidated by Clemens et al. (Clemens and Karsenty, 2011), who demonstrated that insulin signaling can influence the expression of CTSK, a key enzymatic component of osteoclastic bone resorption. These findings suggest that feeding-associated metabolic signals may participate in the establishment of bone resorption rhythms through modulation of essential LRA components. Furthermore, feeding and fasting behaviors are intimately associated with lysosomal biogenesis and autophagy, exhibiting a fundamental pattern in which nutrient abundance suppresses lysosomal biogenesis and autophagic activity, whereas nutrient deprivation enhances both processes (Godar et al., 2015). This relationship provides a more direct mechanistic link between nutritional timing and LRA function and may represent an important pathway through which feeding–fasting cycles influence bone resorption rhythmicity. At the molecular level, under nutrient-replete conditions, mechanistic target of rapamycin complex 1 (mTORC1) is recruited to the lysosomal membrane, where it phosphorylates TFEB and retains it within the cytoplasm, thereby preventing its nuclear translocation and subsequent activation of lysosomal and autophagic gene transcription (Du et al., 2022; Ruolo et al., 2025). Conversely, during nutrient deprivation, mTORC1 dissociates from the lysosomal membrane and loses its ability to phosphorylate TFEB, permitting TFEB nuclear translocation and activation of downstream transcriptional programs that promote lysosomal biogenesis and autophagy to maintain cellular energy homeostasis (Du et al., 2022; Ruolo et al., 2025). Collectively, the “feeding/fasting–mTORC1–TFEB–lysosome” regulatory cascade provides a compelling theoretical framework through which nutritional timing, acting as a peripheral circadian cue, may influence LRA function via modulation of lysosomal biogenesis. Although current investigations of this pathway have largely focused on hepatic physiology and liver-related diseases (Yan, 2022), its potential relevance to skeletal metabolism and osteoclastic lysosomal regulation remains largely unexplored. Elucidating the role of this signaling axis in bone biology may therefore represent a highly innovative and promising direction for future research at the intersection of circadian biology, lysosomal regulation, and skeletal metabolism.

In addition to feeding–fasting cycles, accumulating evidence indicates that systemic redox status also exhibits pronounced circadian rhythmicity and can be transmitted from the core circadian clock to a wide range of peripheral tissues (Fagiani et al., 2022). Previous studies have demonstrated a close association between redox homeostasis and the core clock gene BMAL1 (Zha et al., 2025), with key mediators such as nuclear factor erythroid 2-related factor 2 (Nrf2) and reactive oxygen species (ROS) serving as important links between circadian redox regulation and bone metabolic networks. Nrf2 is a master antioxidant transcription factor whose rhythmic expression is essential for maintaining redox homeostasis (Sutton et al., 2026). Multiple studies have demonstrated reciprocal inhibitory crosstalk between Nrf2 and nuclear factor-κB (NF-κB) (Fagiani et al., 2022; Gao et al., 2022). An early pioneering study revealed that activation of nuclear factor of activated T cells 1 (NFATc1), a major downstream effector of NF-κB signaling, promotes the expression of Atp6v0d2 (Kim et al., 2008). Subsequently, Du et al. (2020) further demonstrated that deficiency of the RNA-binding protein QKI enhances NF-κB signaling and upregulates CTSK expression. Given that Atp6v0d2 is involved in the formation of the lysosomal acidification machinery, whereas CTSK functions as a key proteolytic enzyme responsible for bone matrix degradation, these findings suggest that fluctuations in redox status may regulate the expression and function of LRA-associated molecules through the Nrf2/NF-κB axis, thereby contributing to the control of osteoclastic bone resorption. ROS, a major mediator of oxidative stress, has been widely recognized as an important contributor to the pathogenesis of osteoporosis (Luo et al., 2025). Regarding its relationship with the LRA, in addition to the well-established RANKL–ROS–NF-κB/NFATc1 signaling cascade (Zhou H. et al., 2025), emerging evidence indicates that ROS may also influence lysosomal biogenesis through TFEB. Yang et al. (2025) demonstrated that ROS is essential for TFEB/transcription factor E3 (TFE3)-mediated lysosomal biogenesis. Treatment with the ROS scavenger N-acetyl-L-cysteine (NAC) markedly attenuated TFEB/TFE3 nuclear translocation, lysosomal biogenesis, and autophagic flux. These findings suggest that ROS promotes TFEB/TFE3 nuclear translocation and enhances lysosomal biogenesis, thereby generating rhythmic oscillations that may be functionally coupled to LRA activity.

Physical activity also exhibits characteristic rest–activity cycles that are synchronized with circadian rhythms. The resulting rhythmic mechanical stimuli constitute an important entrainment signal for the skeletal peripheral clock (Gorgun et al., 2026). Direct evidence supporting this concept was provided by Dudek et al. (Dudek et al., 2023), who proposed that circadian clocks periodically integrate responses to mechanical loading and osmotic fluctuations through the phospholipase D2 (PLD2)–mechanistic target of rapamycin complex 2 (mTORC2)–AKT–glycogen synthase kinase 3β (GSK3β) signaling pathway. Osteocytes, the principal mechanosensors embedded within the bone matrix, play a central role in this process by detecting mechanical forces and releasing regulatory factors that indirectly modulate osteoblast and osteoclast activity (Gorgun et al., 2026). More specifically, the mechanosensitive ion channel Piezo1 has been shown to convert extracellular mechanical stimuli sensed by osteocytes, osteoblasts, and bone marrow mesenchymal stem cells into Ca^2+-dependent intracellular signals, thereby activating multiple downstream signaling pathways. These pathways subsequently regulate the OPG/RANKL axis and suppress osteoclastogenesis (Liu C. et al., 2025), ultimately influencing the rhythmic expression and functional activity of key LRA components.

Taken together, the skeletal peripheral clock integrates multiple zeitgeber signals, including feeding–fasting cycles, redox oscillations, and mechanical loading, thereby translating environmental and metabolic information into rhythmic intracellular oscillations within osteoclasts. These convergent signals ultimately act upon the LRA, the terminal effector machinery responsible for bone resorption. Further elucidation of the molecular mechanisms through which peripheral clocks regulate LRA function will provide new theoretical foundations for the development of chronobiology-based precision interventions for osteoporosis (Figure 2).

### 3.3 Cell-autonomous clock

The final level of the circadian timing system, the cell-autonomous clock, represents the smallest functional unit governing circadian rhythms in the body (Mofatteh et al., 2021). Cell-autonomous clocks operate through intracellular clock genes and effector proteins at the molecular level. In mammals, circadian rhythms are driven by a transcription–translation feedback loop (TTFL), which generates approximately 24-h oscillations in gene expression and downstream biological processes (Yuan et al., 2022; Liu Y. et al., 2025). At the core of this loop, the BMAL1/CLOCK heterodimer activates the transcription of clock-controlled genes, whereas cryptochrome (CRY), period (PER), and REV-ERBα/β subsequently inhibit BMAL1/CLOCK activity through negative feedback, thereby establishing self-sustained circadian oscillations (Liu Y. et al., 2025; Rezaeian et al., 2023; Cao X. et al., 2021). Osteoclasts likewise possess an intact TTFL system, which drives the rhythmic expression of LRA-associated molecules.

The rhythmic execution of bone resorption by the LRA depends on the oscillatory expression of its molecular and protein components, a process regulated by cell-autonomous clock genes. Schilperoort et al. (2020) demonstrated that, in murine osteoclasts (notably, mice are nocturnal animals, such that their active phase corresponds approximately to the human daytime), not only core clock genes including Bmal1, Per1, and Per2, but also CTSK, a hallmark marker of the osteoclastic LRA, exhibit significant circadian rhythmicity. Specifically, BMAL1 and CTSK display a day-high/night-low expression pattern, REV-ERBα peaks subsequent to BMAL1, whereas Per1 and Per2 exhibit the opposite pattern, with higher expression during the night. These findings provide direct molecular evidence that an intrinsic osteoclastic clock contributes to the rhythmic regulation of the LRA. Among clock components, BMAL1 appears to be particularly closely linked to LRA function. Previous studies have demonstrated that BMAL1 can directly bind to the CTSK promoter and drive its rhythmic transcription (Fujihara et al., 2014). In addition, BMAL1 cooperates with CLOCK to enhance the transcriptional expression of nuclear factor of activated T cells 1 (NFATc1) (Xu et al., 2016), a master regulator of osteoclastogenesis that is directly associated with the LRA acidification component Atp6v0d2 (Kim et al., 2008) and the bone resorption effector CTSK (Costa et al., 2011). These findings provide a mechanistic basis by which the BMAL1/CLOCK complex promotes LRA activity and contributes to the characteristic nocturnal predominance of bone resorption. Notably, although direct evidence linking BMAL1 to osteoclastic lysosomal biogenesis remains limited, studies in the central nervous system have demonstrated that core clock components can regulate the autophagy–lysosome pathway (McKee et al., 2023). This observation suggests that BMAL1 may also participate in the regulation of LRA functional rhythmicity through lysosome-related mechanisms, a possibility that warrants further investigation. Conversely, owing to its inhibitory effects on the BMAL1/CLOCK complex, REV-ERBα appears to exert an opposing influence on LRA activity. A recent study showed that the REV-ERBα agonist SR9009 significantly suppresses RANKL signaling (Zhang et al., 2025), resulting in reduced expression of TFEB, CTSK, and Atp6v0d2 and consequently attenuating LRA-mediated bone resorption. This inhibitory effect, occurring subsequent to BMAL1 activation, may contribute to the circadian phase characterized by relatively lower bone resorption activity during the resting period.

Interestingly, in addition to the canonical BMAL1/CLOCK-centered circadian pathway, cell-autonomous rhythmicity also involves parallel regulatory axes. Previous studies have shown that TFEB/TFE3 can directly bind to the promoter region of NR1D1 (REV-ERBα), thereby regulating circadian gene expression through a mechanism that operates in parallel with, and independently of, the core molecular clock machinery (Pastore and Ballabio, 2019). This finding suggests that the relationship between the cell-autonomous clock and the LRA is not simply a linear upstream–downstream hierarchy but may instead constitute a bidirectionally regulated circadian module. Within this framework, the clock machinery drives the rhythmic expression of key LRA components, whereas LRA-associated transcriptional networks can, in turn, participate in the stabilization and fine-tuning of circadian oscillations. Together, these reciprocal interactions contribute to the temporal organization of osteoclastic bone resorption.

Collectively, as the final layer through which circadian signals regulate LRA rhythmicity, the cell-autonomous clock exhibits a bidirectional regulatory relationship with the osteoclastic LRA. The TTFL provides the mechanistic foundation for positive circadian regulation of LRA-associated genes, while LRA-related signaling and transcriptional components can reciprocally influence key elements of the TTFL through parallel regulatory pathways. This intricate and highly coordinated feedback network constitutes the fundamental molecular framework underlying circadian control of LRA function and ensures the temporal precision of osteoclastic bone resorption (Figure 2).

## 4 Circadian dysregulation contributes to the pathophysiology of osteoporosis

### 4.1 Postmenopausal osteoporosis (PMOP)

PMOP is a bone metabolic disorder primarily driven by decreased estrogen levels, particularly 17β-estradiol (E2) levels, characterized by excessive OC activity and consequent bone loss (Luo et al., 2025; Shi et al., 2015; Park et al., 2021). However, hormone levels are susceptible to perturbations in circadian synchrony, which can influence bone metabolism and further affect skeletal repair and remodeling. In OCs, estrogen deficiency induces upregulation of receptor activator of RANKL, promoting OC differentiation and activity, ultimately resulting in bone loss (Cheng et al., 2022; Park et al., 2021). The interplay between intrinsic hormonal changes and extrinsic circadian disruptions represents a key chronobiological mechanism underlying PMOP.

Circadian disruption can interfere with hormone regulation. Sijie Fan et al. demonstrated that 3-methyl-4-nitrophenol disrupts clock gene expression, thereby impairing early follicular development in murine ovaries (Fan et al., 2022). Similarly, RongJin Lin et al. reported that sleep deprivation modulates circadian gene expression in pregnant rats via the PI3K/Akt signaling pathway, subsequently affecting sex hormone secretion (Lin and Dai, 2022). Under physiological conditions, estrogen regulates bone metabolism directly or indirectly via estrogen receptors (ERs) (Lu and Tian, 2023). Specifically, E2 binding to ERα inhibits RANKL-induced cytoskeletal remodeling, forming an ERα/Src homology 2 domain-containing phosphatase 2 (SHP2)/cellular Src kinase (c-Src) complex that attenuates bone resorption (Park et al., 2021). Moreover, estrogen transcriptionally activates E2/ERα signaling, enhancing levels of transient receptor potential vanilloid 5 (TRPV5) to further suppress OC-mediated bone resorption (Song T. et al., 2018).

Hormonal fluctuations not only disrupt circadian synchrony but also directly influence bone metabolism. In postmenopausal women, low E2 levels trigger circulating macrophages to release pro-inflammatory cytokines such as interleukin-1 (IL-1), interleukin-4 (IL-4), IL-6, and TNF-α, which in turn stimulate RANKL production, activate OCs, and exacerbate trabecular bone loss (Cheng et al., 2022; Park et al., 2021; Hsu et al., 2024; Szulc et al., 2006). Consequently, circadian dysregulation can precipitate abnormal estrogen levels, promoting bone resorption and contributing to osteoporosis.

Under normal conditions, lysosomal function exhibits circadian rhythmicity, with fluctuations in acidification and enzymatic activity across the day–night cycle (Aviram et al., 2021). Nazma Malik et al. demonstrated that AMP-activated protein kinase (AMPK) phosphorylation can induce lysosomal and mitochondrial biogenesis (Malik et al., 2023). Estrogen, via ERα, modulates AMPK activity, indirectly regulating OC function and lysosomal enzyme expression (Gandhi et al., 2021). Therefore, in PMOP, decreased E2 levels impair lysosomal function, disrupting normal bone resorption and accelerating bone loss.

**FIGURE 3:**
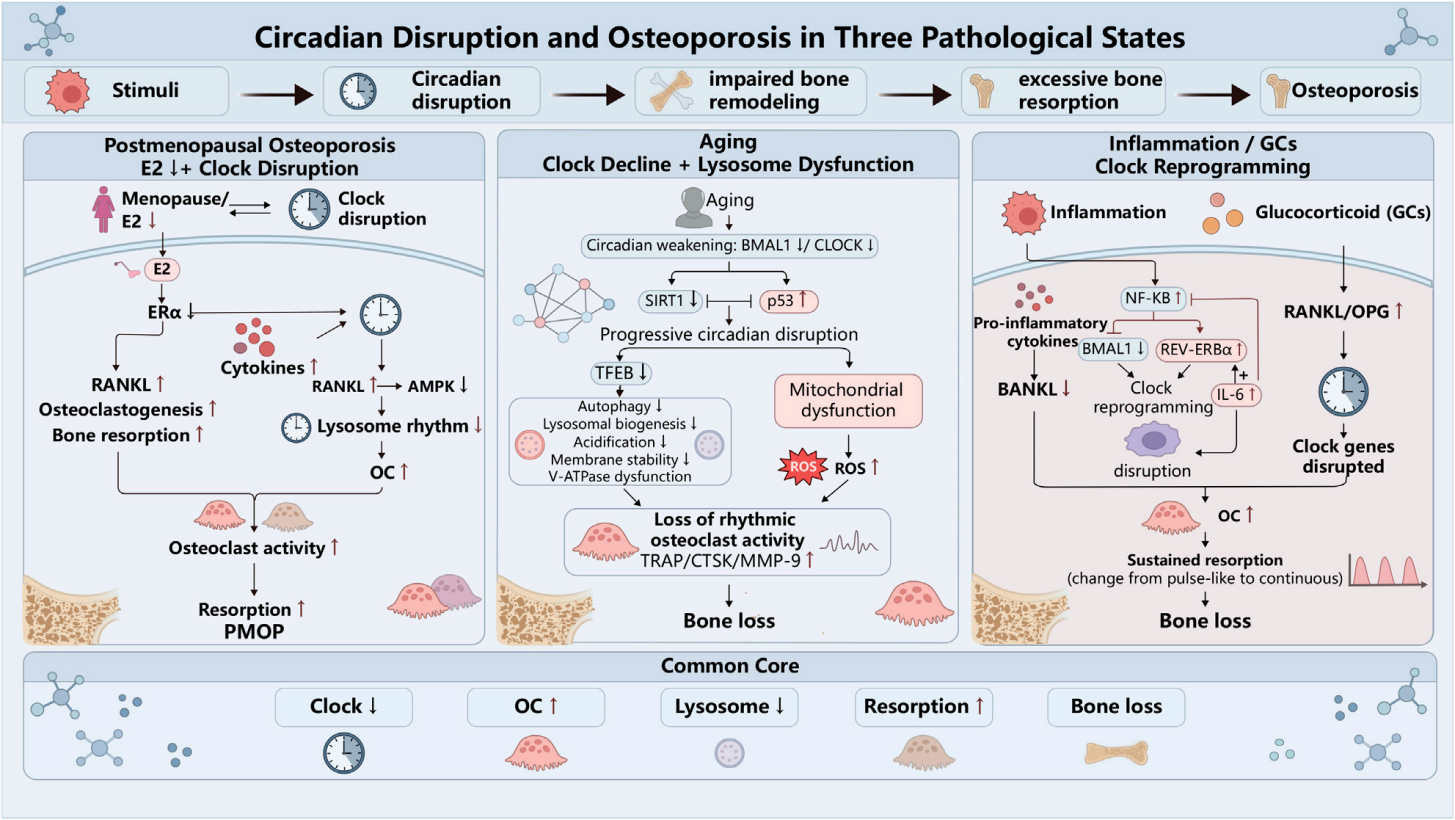
Circadian Disruption Drives Osteoporosis in Postmenopause, Aging, and Inflammatory/Glucocorticoid States. Circadian disruption drives osteoporosis via osteoclast (OC) overactivation or dysregulation across postmenopause, aging, and inflammatory/glucocorticoid states, altering RANKL signaling, lysosome function, and rhythmic bone resorption. Common core mechanisms include clock downregulation, impaired lysosomal activity, enhanced OC resorption, and bone loss. Abbreviations: AMPK, AMP-activated protein kinase; E2, 17β-estradiol; ERα, estrogen receptor alpha; IL-6, interleukin-6; NF-κB, nuclear factor kappa B; OC, osteoclast; OPG, osteoprotegerin; PMOP, postmenopausal osteoporosis; REV-ERBα, nuclear receptor subfamily 1 group D member 1; SIRT1, sirtuin 1; TRAP, tartrate-resistant acid phosphatase.

In summary, postmenopausal estrogen deficiency not only directly promotes OC hyperactivation and bone loss but also disrupts circadian rhythms and lysosomal function, amplifying abnormal bone resorption. This multifactorial mechanism underlies the pathogenesis of PMOP (Figure 3).

### 4.2 Senile osteoporosis

Senile osteoporosis is an age-related bone metabolic disorder, typically accompanied by reduced bone turnover and impaired circadian clock function (Brown, 2017; Gossiel et al., 2018). With aging, circadian disruption not only perturbs OC function but also diminishes lysosomal “efficiency,” further exacerbating imprecise bone resorption and resulting in region-specific bone loss.

Core circadian clock molecules, including BMAL1 and CLOCK, exhibit circadian rhythmicity in bone metabolic cells, directly or indirectly regulating OC differentiation and activity. Reduced BMAL1 expression impairs circadian control over OC activity, leading to dysregulated bone resorption (Xu et al., 2016). This phenomenon can be explained through multiple mechanisms. First, circadian dysfunction decreases lysosomal “apparatus efficiency” (Song et al., 2018b). Attenuated expression and rhythmicity of BMAL1 and CLOCK disrupt downstream regulatory factors, particularly transcription factor EB (TFEB) (Pastore and Ballabio, 2019). TFEB is a central regulator of aging and age-related diseases, positively controlling autophagy and lysosomal biogenesis (Abokyi et al., 2023; Song et al., 2021). Reduced TFEB activity suppresses lysosomal biogenesis and autophagy, leading to insufficient lysosomal acidification, compromised membrane stability, and impaired function of membrane proteins such as V-ATPase (Abokyi et al., 2023; Song et al., 2021; Pan and Valapala, 2023; Wang et al., 2024).

Simultaneously, circadian disruption perturbs the diurnal oscillation of ROS. Aging-associated cellular senescence, coupled with diminished autophagy, leads to mitochondrial dysfunction and ROS accumulation, thereby reducing osteogenic activity, compromising lysosomal membrane integrity, and promoting OC differentiation (Wang J. et al., 2023; Zheng et al., 2026; Agidigbi and Kim, 2019). Consequently, OC activity becomes abnormally enhanced, bone resorption accelerates and loses spatial precision, and bone matrix damage increases. Overall, lysosomal function in OCs declines progressively, manifesting as impaired acidification and membrane homeostasis, resulting in OC dysfunction and reduced accuracy in bone resorption and bone metabolic balance (Jiang et al., 2024; Pu et al., 2016; Platt et al., 2018; Colacurcio and Nixon, 2016).

Furthermore, lysosomal impairment caused by circadian disruption directly affects OC activity and resorption precision. Declines in TFEB activity and ROS accumulation enhance OC activity while reducing lysosomal function. During this process, OCs accelerate bone matrix degradation by upregulating tartrate-resistant acid phosphatase (TRAP), CTSK, and MMP9 expression, leading to bone mass loss and diminished resorption efficiency (Takayanagi et al., 2002; Teitelbaum and Ross, 2003). Sirtuin 1 (SIRT1), serving as a key mediator linking the circadian clock and lysosomal function, regulates TFEB activity and suppresses ROS accumulation to stabilize lysosomal function; its deficiency or reduced activity further exacerbates OC hyperactivation (Masri et al., 2014; Chang and Guarente, 2013; Qu et al., 2018; Mao et al., 2020; Yau et al., 2019; Chen et al., 2022; Du et al., 2025). Additionally, tumor protein p53 (p53) indirectly contributes to circadian disruption-mediated lysosomal dysfunction by blocking CLOCK-BMAL1 binding to the PER2 promoter (Miki et al., 2013).

In summary, in senile osteoporosis, aging-related circadian dysregulation affects OC lysosomal function through key factors including TFEB, SIRT1, ROS, and p53, resulting in abnormal enhancement of bone resorption activity and imprecise localization, ultimately accelerating bone matrix damage and bone mass loss. This mechanism provides a novel perspective on the pathophysiology of osteoporosis (Figure 3).

### 4.3 Inflammation-related osteoporosis

The development of inflammation-related osteoporosis involves bidirectional interactions between pro-inflammatory cytokines and the circadian regulation of bone metabolism. Pro-inflammatory mediators such as TNF-α and IL-1β disrupt the skeletal circadian clock, aberrantly enhance osteoclast activity, and accelerate bone resorption and bone loss. Inflammatory cytokines influence bone resorption by perturbing circadian regulation, thereby disrupting normal bone metabolic rhythms. Acting as “rectifiers,” these cytokines can lock bone metabolism into a sustained resorptive state. Under inflammatory conditions, elevated levels of TNF-α, interleukins (IL-1, IL-6, interleukin-17 (IL-17)), and other cytokines stimulate osteoblasts to upregulate RANKL, promoting OC differentiation and enhancing bone resorption, resulting in bone loss (Cheng et al., 2022; Kitaura et al., 2020; Ohnuma et al., 2019; Zhao, 2020). For instance, TNF-α directly upregulates RANKL expression in osteoblasts, facilitating OC formation (Marahleh et al., 2019), while IL-17 promotes mesenchymal stem cells (MSCs) toward osteogenic differentiation, indirectly increasing OC differentiation (Fischer and Haffner-Luntzer, 2022). Pro-inflammatory cytokines, particularly TNF-α and IL-1β, also interfere with circadian regulation at the molecular level. They activate pathways such as nuclear factor kappa B (NF-κB) (Yan et al., 2023), suppress BMAL1 expression, and upregulate its inhibitor, the nuclear receptor REV-ERBα (Chen et al., 2020), effectively “reprogramming” the circadian machinery of immune and bone cells toward a pro-resorptive state. NF-κB signaling is a well-established mediator of abnormal bone metabolism (Abu-Amer, 2013), critical for RANKL-induced OC generation; inhibiting NF-κB effectively reduces OC formation and bone resorption (Abu-Amer, 2013; Zeng et al., 2016). Genetic deletion or mutation of RANKL or RANK can cause severe bone dysplasia, resulting in complete OC deficiency (Lacey et al., 1998; Dougall et al., 1999). Moreover, NF-κB signaling and core circadian loops exhibit mutual inhibition, creating a vicious cycle that sustains inflammation and circadian disruption. Circadian clocks also regulate immune function (Zhao et al., 2025); for example, disruption of PER1/2 impairs diurnal rhythmicity, enhancing macrophage pro-inflammatory activation (Xu et al., 2014). Low BMAL1 expression is likewise associated with elevated IL-6 levels (Tang et al., 2022). Hence, circadian dysregulation amplifies inflammatory cytokine expression, accelerating bone loss and contributing to inflammation-associated osteoporosis.

Collectively, circadian disruption promotes the production of pro-inflammatory cytokines, which enhance RANKL expression, activate NF-κB signaling, and reprogram the molecular clocks of skeletal and immune cells. These alterations result in sustained osteoclast activation and excessive bone resorption, ultimately contributing to the development of inflammation-related osteoporosis.

### 4.4 Glucocorticoid-induced osteoporosis

As important endocrine circadian signals (Martinez et al., 2024), glucocorticoids exert profound effects on skeletal homeostasis. Dysregulation of glucocorticoid signaling not only directly influences osteoclast differentiation and function but also exacerbates bone metabolic imbalance by disrupting the circadian regulatory network of the skeletal system, thereby contributing to the development of osteoporosis.

The pathogenesis of glucocorticoid-induced osteoporosis can be explained from two complementary perspectives. First, excessive glucocorticoid exposure increases the RANKL/OPG ratio, thereby promoting osteoclast maturation, activation, and bone resorption (Cheng et al., 2022; Urquiaga and Saag, 2022). Concurrently, reduced expression of OPG creates a microenvironment favorable for osteoclastogenesis, while glucocorticoids further enhance bone resorption by prolonging osteoclast survival (Seibel et al., 2013). Second, glucocorticoids function as key circadian hormones whose secretion follows a robust daily rhythm, with peak concentrations closely associated with the onset of the active phase (Jones et al., 2021; Shimba et al., 2021; Dickmeis, 2009). Through regulation of clock components such as PER1, PER2, REV-ERBα, and nuclear factor interleukin-3-regulated protein (NFIL3), glucocorticoids play a critical role in synchronizing peripheral circadian rhythms (Dickmeis, 2009; Shimba and Ikuta, 2020). However, prolonged exogenous glucocorticoid administration can disrupt these physiological timing signals, leading to circadian misalignment and desynchronization of the skeletal clock network. Such disturbances further aggravate osteoclastic bone resorption and accelerate bone loss. Consequently, the combined disruption of circadian homeostasis and skeletal remodeling results in a pronounced imbalance between bone resorption and bone formation, increasing fracture susceptibility and exacerbating the clinical manifestations of osteoporosis.

Collectively, glucocorticoids contribute to osteoporosis through both direct and indirect mechanisms. By increasing the RANKL/OPG ratio, prolonging osteoclast survival, and disrupting the expression of circadian clock genes, glucocorticoids synergistically enhance bone resorption and skeletal deterioration, thereby playing a central role in the initiation and progression of osteoporosis (Figure 3).

## 5 Therapeutic strategies for osteoporosis based on circadian rhythm reprogramming

### 5.1 Chronopharmacology: optimal timing for anti-resorptive agents

With increasing understanding of circadian regulation in osteoporosis, research exploring how diurnal rhythms influence drug efficacy has gained momentum, with a primary focus on leveraging circadian biology for therapeutic intervention (Song et al., 2018b; Qian et al., 2025). Effective osteoporosis treatment not only depends on the pharmacological properties of drugs but also on their bioavailability and the circadian rhythmicity of bone matrix degradation. Anti-resorptive agents, such as bisphosphonates (BPs) and Denosumab, exhibit robust clinical efficacy; however, their therapeutic outcomes are often influenced by the timing of administration (Drake et al., 2008; Roane et al., 2023; Lee et al., 2026; Wang LT. et al., 2023). Thus, optimizing the dosing schedule according to the circadian rhythm of bone matrix turnover may enhance treatment efficacy.

Under physiological conditions, bone matrix degradation displays diurnal rhythmicity (Darling et al., 2025; Luo et al., 2021; Kikyo, 2024; Ze et al., 2025). Peak bone resorption typically occurs at night (Darling et al., 2025), coinciding with heightened OC activity (Darling et al., 2025) and maximal lysosomal acidification (Aviram et al., 2021; Mazzoccoli et al., 2015). BPs inhibit OC activity by targeting farnesyl pyrophosphate synthase (FPPS), disrupting the cytoskeleton and inducing OC apoptosis (Drake et al., 2008; Roane et al., 2023; Lee et al., 2026; Wang LT. et al., 2023). Denosumab, a monoclonal antibody against RANKL, effectively suppresses OC-mediated bone resorption. Both are primary pharmacological agents for osteoporosis treatment (Drake et al., 2008; Roane et al., 2023; Lee et al., 2026; Wang LT. et al., 2023). Based on the above circadian rhythm-related evidence, several studies have theoretically proposed that drug exposure levels may exhibit temporal associations with fluctuations in bone resorption activity (Winter et al., 2021; Dallmann et al., 2016). However, this hypothesis remains insufficiently supported by robust clinical evidence and therefore does not yet justify the formulation of definitive chronotherapeutic dosing recommendations in clinical practice (Winter et al., 2021; Dallmann et al., 2016).

For oral bisphosphonates, their clinical administration is typically recommended in the fasting state upon waking, primarily due to their low oral bioavailability and the risk of esophageal irritation, thereby optimizing absorption efficiency and minimizing adverse gastrointestinal effects (Fuggle et al., 2022; Lin, 1996). In addition, bisphosphonates exhibit a high affinity for bone mineral and can be stably incorporated into bone tissue, where they may persist for extended periods, resulting in sustained anti-resorptive effects lasting from months to years (Gehrke et al., 2023; Reid and Billington, 2022). Consequently, morning administration may still, to some extent, provide pharmacological coverage during the nocturnal period when bone resorption activity is relatively elevated. In contrast, Denosumab, a monoclonal antibody targeting RANKL (Drake et al., 2008; Roane et al., 2023; Lee et al., 2026; Wang LT. et al., 2023), exerts its pharmacological effects primarily through systemic inhibition of RANKL signaling (Hanley et al., 2012). Mechanistically, its activity is not directly governed by circadian fluctuations in bone resorption dynamics. Therefore, its dosing schedule is relatively flexible and not strictly constrained by circadian timing considerations.

Drug bioavailability is also subject to circadian influence (Du et al., 2023; Seto et al., 2020). Gastrointestinal (GI) circadian rhythms regulate gastric acid secretion, GI motility, and drug absorption (Bishehsari et al., 2025). Gastric acid production increases throughout the day and peaks during late night and early morning, while gastric pH reaches its nadir at night (Moore and Halberg, 1986; Moore et al., 1992). These fluctuations significantly affect the bioavailability of orally administered drugs, as GI circadian rhythms directly modulate absorption efficiency (Seto et al., 2020). Animal studies indicate that oral drug absorption can vary significantly depending on administration timing (Seto et al., 2020). Additionally, pharmacokinetic parameters—including plasma concentration, clearance, and both free and total drug bioavailability—differ markedly across different dosing times (Engli et al., 1983). Therefore, the timing of oral administration should consider GI circadian rhythms to maximize drug absorption and overall bioavailability. Integrating chronopharmacology with osteoporosis therapy enables the identification of an optimal dosing window for anti-resorptive agents. This window should correspond to peak bone resorption, i.e., nighttime administration, which not only aligns with heightened OC activity but also enhances drug efficacy by effectively suppressing excessive bone resorption. Consequently, this approach may optimize therapeutic outcomes and improve patient experience.

In conclusion, precision dosing guided by circadian biology represents a promising strategy for optimizing osteoporosis therapy. Future research should further investigate the temporal efficacy of different anti-resorptive agents and incorporate individual patient circadian variability to develop personalized treatment regimens (Table 1).

**TABLE 1:** Circadian rhythm–based therapeutic strategies for osteoporosis.

Category	Strategy/Target	Circadian rationale	Mechanism/Observation	Potential timing or intervention strategy	Evidence status/Notes	References
Clinical anti-resorptive therapy	Oral bisphosphonates: fasting administration	Oral bisphosphonate absorption is strongly affected by food, beverages, and posture-related safety issues	Extremely low oral bioavailability; risk of esophageal irritation if dosing instructions are not followed	Morning administration after rising, with plain water; avoid food, other drugs, and lying down for at least 30 min	Established clinical administration rule; mainly pharmacokinetic and safety-based rather than purely circadian	Fuggle et al. (2022); Lin (1996)
Clinical anti-resorptive therapy	Oral bisphosphonates: sustained skeletal reservoir	Bone resorption shows diurnal rhythmicity and tends to increase during nighttime or fasting periods	Bisphosphonates bind strongly to bone mineral and exert prolonged anti-resorptive effects after skeletal incorporation	Morning dosing can still maintain sufficient skeletal drug reservoir to cover nocturnal resorption activity	Established clinical therapy; circadian coverage is a mechanistic inference based on long skeletal retention	Gehrke et al. (2023), Reid and Billington (2022)
Clinical anti-resorptive therapy	Bisphosphonate mechanism	Osteoclast-mediated matrix degradation is a major effector of rhythmic bone resorption	FPPS inhibition disrupts osteoclast cytoskeletal organization and promotes osteoclast apoptosis	Suppression of osteoclast activity across the bone remodeling cycle	Established anti-resorptive mechanism	Drake et al. (2008), Roane et al. (2023), Lee et al. (2026), Wang et al. (2023d)
Clinical anti-resorptive therapy	Denosumab	RANKL-dependent osteoclastogenesis contributes to excessive bone resorption	Monoclonal antibody against RANKL; inhibits osteoclast formation, function, and survival	Dosing is relatively independent of daily bone turnover timing; strict maintenance of the dosing interval is more important	Established clinical therapy; recommended as 60 mg subcutaneously every 6 months	Hanley et al. (2012)
Chronopharmacology	Gastrointestinal circadian pharmacokinetics	GI secretion, motility, pH, and absorption fluctuate across the day	Circadian variation may influence plasma concentration, clearance, and oral bioavailability	Oral dosing schedules should consider GI circadian rhythms, especially for drugs with low or food-sensitive absorption	General chronopharmacological principle; direct osteoporosis-specific evidence remains limited	Du et al. (2023), Seto et al. (2020)
Experimental clock-gene modulation	REV-ERBα activation: clock restoration	REV-ERBα is a core clock component involved in maintaining osteoclast rhythmicity	REV-ERBα activation may reinforce osteoclast circadian oscillation	Experimental REV-ERBα agonist-based chronotherapy, such as SR9009-mediated intervention	Preclinical strategy; not established for clinical osteoporosis treatment	Wang et al. (2023e)
Experimental clock-gene modulation	REV-ERBα activation: anti-resorptive effect	Osteoclast clock disruption may promote excessive differentiation and bone resorption	REV-ERBα activation suppresses osteoclast hyper-differentiation and attenuates bone loss	Circadian clock-targeted intervention to reduce excessive bone resorption	Preclinical evidence; clinical translation remains uncertain	Song et al. (2018c), Liu et al. (2020)
Experimental clock-gene modulation	CRY stabilization	CRY proteins are core negative regulators of the circadian transcriptional feedback loop	Stabilizing CRY protein levels may reinforce core clock oscillation and restore physiological remodeling rhythms	Pharmacological CRY stabilization as a potential clock-repair strategy	Experimental/potential strategy; bone-specific evidence remains limited	Deng et al. (2023)
Experimental clock-gene modulation	BMAL1-axis regulation	BMAL1 is a central oscillator implicated in osteoclast differentiation and resorptive activity	BMAL1 downregulation is associated with osteoclast hyper-differentiation and excessive bone resorption	Indirect modulation through ROR agonists or REV-ERB antagonists may enhance BMAL1 rhythmicity	Promising but mostly preclinical; no direct BMAL1-stabilizing small molecule has been established	Yin et al. (2024), Fan et al. (2026), Dadon-Freiberg et al. (2021)
Experimental clock-gene modulation	BMAL1 and lysosomal resorptive rhythm	Osteoclast intrinsic clocks may gate lysosomal acidification and resorptive apparatus activity	BMAL1 stabilization may limit abnormal lysosomal remodeling and preserve rhythmic LRA activity	Restore osteoclast-intrinsic clock function to normalize matrix degradation	Mechanistic inference requiring further validation	Xie et al. (2022)
Lysosomal homeostasis restoration	TFEB activation	Lysosomal function and osteoclast resorptive activity exhibit rhythmic regulation	TFEB promotes lysosomal biogenesis, quality control, and maintenance of the osteoclast resorptive apparatus	Potential activation of TFEB during periods of heightened lysosomal demand	Experimental strategy; optimal timing requires further validation	Lacombe et al. (2013)
Lysosomal homeostasis restoration	Quercetin/resveratrol	Natural compounds may influence autophagy–lysosome pathways and interact with circadian regulation	Quercetin and resveratrol have been reported to enhance TFEB-related lysosomal function and degradation capacity	Rhythmically timed administration may theoretically improve lysosomal quality control	Potential adjunctive strategy; direct chronotherapeutic evidence in osteoporosis remains insufficient	An and Hu (2022), Chen et al. (2021b), Shao et al. (2021)
Lysosomal acidification control	V-ATPase targeting	V-ATPase-mediated acidification supports osteoclast lysosomal function and matrix degradation	Overactive V-ATPase is associated with osteoclast hyperactivity and intensified bone resorption	Selective, temporally coordinated suppression of osteoclast acidification may reduce nocturnal resorption peaks	Experimental strategy; systemic V-ATPase inhibition raises safety and specificity concerns	Futai et al. (2019), Duan et al. (2018), Chu et al. (2021), Bebas et al. (2009)
Central clock entrainment	Timed light exposure	Light is the dominant environmental cue for SCN entrainment	Daytime light exposure may synchronize central circadian rhythms and support vitamin D-related skeletal metabolism	Regular daytime light exposure; avoidance of inappropriate nocturnal light exposure	Lifestyle-based adjunctive strategy	Lucassen et al. (2016), Yuan et al. (2026), Bikle (2025), Carlberg et al. (2023), Makida et al. (2020), Ochiai et al. (2021)
Hormonal circadian regulation	Melatonin supplementation	Melatonin is regulated by the SCN and environmental light	Melatonin may promote bone formation and inhibit bone resorption	Nighttime supplementation and avoidance of nighttime light exposure may help restore circadian bone metabolic regulation	Potential adjunctive strategy; clinical dosing and long-term skeletal outcomes require further validation	Yang et al. (2022), Li et al. (2019), Touitou et al. (2017)
Peripheral clock synchronization	Timed exercise	Mechanical loading effects on bone may be modulated by circadian phase and metabolic state	Properly timed exercise may enhance osteogenic responses and improve bone remodeling balance	Postprandial exercise may be beneficial; some evidence suggests stronger anabolic responses at specific daytime windows	Lifestyle intervention; exercise intensity should be individualized according to fracture risk	Borer et al. (2019)
Mechanical loading intervention	Whole-body vibration/high-impact exercise	Mechanical stimulation can entrain peripheral skeletal responses and promote bone formation	High-impact exercise and whole-body vibration increase skeletal loading and may improve BMD	Use as adjunctive intervention under individualized risk assessment and supervision	High-impact exercise may be inappropriate for severe osteoporosis or high fracture-risk patients	Qian et al. (2025)
Metabolic clock synchronization	Timed feeding	Feeding time is a major cue for peripheral clocks and metabolic gene rhythmicity	Aligning feeding windows with circadian rhythms may regulate metabolic and bone-related genes	Synchronize feeding windows with daytime activity and metabolic rhythms; avoid chronically irregular eating patterns	Lifestyle-based adjunctive strategy; direct osteoporosis-specific clinical evidence remains limited	Qian et al. (2025)

Abbreviations: BMAL1, brain and muscle ARNT-like protein 1; CLOCK, circadian locomotor output cycles kaput; CRY, cryptochrome; REV-ERBα (NR1D1), nuclear receptor subfamily 1 group D member 1; ROR, retinoic acid receptor-related orphan receptor; SCN, suprachiasmatic nucleus; RANKL, receptor activator of nuclear factor κB ligand; FPPS, farnesyl pyrophosphate synthase; TFEB, transcription factor EB, a master regulator of lysosomal biogenesis; LRA, lysosomal–resorptive apparatus; V-ATPase, vacuolar-type H^+^-ATPase; GI, gastrointestinal; BMD, bone mineral density.

### 5.2 Clock gene modulators: targeting the “gatekeepers” of bone metabolism

The circadian clock, as a pivotal mechanism for maintaining cellular function and rhythmicity, plays a critical role in bone metabolism. OCs, as key regulators of bone homeostasis, are highly sensitive to circadian disruption, which is closely associated with osteoporosis and related disorders. Core oscillators, such as REV-ERBα, cryptochrome (CRY), and BMAL1, are essential for maintaining the rhythmicity of OCs. Modulating the activity of these clock genes can restore normal OC function. By activating core oscillators or stabilizing BMAL1 expression, it is possible not only to repair the circadian clock within OCs but also to attenuate their excessive differentiation and bone resorptive activity, providing a novel therapeutic strategy for osteoporosis.

The core circadian clock comprises multiple genes, among which REV-ERBα, CRY, and BMAL1 act as central oscillators that regulate the cell cycle and functional homeostasis (Schrader et al., 2024; Bolsius et al., 2021). Studies indicate that OCs in osteoporosis frequently exhibit circadian dysregulation, resulting in hyperactive differentiation and resorptive activity (Xu et al., 2016). Therefore, targeting these core oscillators to restore the OC intrinsic clock can normalize circadian rhythms, reduce excessive OC differentiation, and correct disordered bone resorption. For instance, SR9009, a REV-ERBα agonist, enhances REV-ERBα activity and restores OC circadian rhythmicity (Wang R. et al., 2023). Extensive animal studies have demonstrated that SR9009 effectively suppresses OC hyper-differentiation and mitigates bone resorption (Song et al., 2018c; Liu et al., 2020). Additionally, CRY stabilizers have been shown to stabilize CRY protein levels in OCs, thereby repairing the circadian clock and promoting the physiological rhythm of bone metabolism (Deng et al., 2023).

BMAL1, another core oscillator (Schrader et al., 2024; Bolsius et al., 2021), plays a crucial role in OCs (Ze et al., 2025). In osteoporosis, BMAL1 expression is often downregulated, which correlates with enhanced OC differentiation and hyperactive bone resorption (Yin et al., 2024). However, no small-molecule compounds capable of directly binding to and stabilizing BMAL1 have been identified to date. Nevertheless, accumulating evidence suggests that modulation of upstream regulators of BMAL1, such as retinoic acid receptor-related orphan receptor (ROR) agonists and REV-ERB antagonists, can enhance BMAL1 expression and reinforce its circadian rhythmicity (Fan et al., 2026; Dadon-Freiberg et al., 2021). By restoring osteoclastic clock function, these approaches may prevent aberrant remodeling of the lysosomal apparatus and preserve the physiological rhythmicity of LRA activity (Xie et al., 2022). Evidence suggests that BMAL1 stabilization improves OC functionality and attenuates excessive resorptive activity (Xie et al., 2022). Certain small molecules, including specific plant-derived compounds or synthetic agents, can modulate BMAL1 activity, thereby restoring bone metabolic balance, limiting overactive lysosomal acidification, suppressing OC hyper-differentiation, and normalizing bone matrix turnover.

In summary, targeting clock genes—particularly core oscillators and the BMAL1 axis—represents an effective strategy to restore normal circadian function in OCs, thereby reducing excessive bone resorption. Future studies should further explore the clinical applicability of clock gene modulators, providing more precise and effective interventions for the management of osteoporosis (Table 1).

### 5.3 Lysosomal homeostasis restoration: reconstructing the “resorptive apparatus” barrier

In the pathogenesis of osteoporosis, lysosomes, as the “resorptive apparatus” of OCs, play a pivotal role. Lysosomes not only serve as the primary site for bone matrix degradation by OCs, but their proper functionality directly regulates bone resorption. However, under osteoporotic conditions, lysosomal function is often compromised, characterized by insufficient acidification and reduced degradative efficiency. This dysfunction contributes to hyperactive OCs, exacerbates matrix degradation, and ultimately leads to bone loss. Therefore, restoring lysosomal homeostasis, particularly in terms of lysosomal biogenesis and functional regulation, represents a critical therapeutic strategy for osteoporosis.

TFEB has recently been identified as a master transcriptional regulator of lysosomal biogenesis (Lacombe et al., 2013). Evidence indicates that TFEB is highly expressed in OCs and serves as a key regulator of lysosomal formation (Lacombe et al., 2013). In pathological states, however, TFEB expression is often suppressed, resulting in lysosomal dysfunction and enhanced bone resorption. Consequently, temporally targeted interventions aimed at the TFEB pathway can effectively restore lysosomal biogenesis and quality control, thereby modulating OC “resorptive apparatus” functionality. Moreover, given that lysosomal activity exhibits circadian rhythmicity (Aviram et al., 2021; Mazzoccoli et al., 2015), activating TFEB during periods of peak lysosomal function may maximize quality control and restore normal bone matrix degradation. Natural compounds such as quercetin and resveratrol have been shown to activate TFEB signaling and enhance lysosomal biogenesis (An and Hu, 2022; Chen M. et al., 2021; Shao et al., 2021). Through temporally optimized administration, these compounds not only augment lysosomal function but also improve therapeutic outcomes in osteoporosis. Hence, aligning drug delivery with lysosomal circadian rhythm and TFEB activity can enhance the efficacy of these interventions.

V-ATPase is the core enzyme responsible for lysosomal acidification. In patients with osteoporosis, overactive V-ATPase is closely associated with OC hyper-differentiation and intensified bone resorption (Tsukuba et al., 2017; Qin et al., 2025; Futai et al., 2019; Duan et al., 2018; Chu et al., 2021). V-ATPase activity exhibits marked circadian rhythmicity (Bebas et al., 2009), typically peaking at night, coinciding with the peak of bone matrix degradation. Therefore, rhythmically targeting V-ATPase inhibition can effectively mitigate nocturnal resorptive peaks. For example, small-molecule drugs or natural compounds administered in a temporally coordinated manner can suppress V-ATPase activity during the night, thereby preventing excessive OC activation and matrix degradation.

In summary, restoring lysosomal homeostasis is crucial for regulating the functionality of the OC “resorptive apparatus.” Temporally targeted interventions via the TFEB pathway, activation of TFEB by natural compounds, and rhythmic inhibition of V-ATPase can collectively modulate OC-mediated bone resorption. These strategies provide a novel avenue for therapeutic intervention in osteoporosis (Table 1).

### 5.4 Environmental and lifestyle interventions: reestablishing central–peripheral synchrony

In modern society, irregular lifestyles, insufficient physical activity, and limited light exposure are common, leading to widespread circadian disruption, which negatively impacts skeletal health. Therefore, interventions such as light therapy and rhythmically timed exercise, aimed at restoring synchrony between central and peripheral clocks, may provide novel approaches for osteoporosis management.

In osteoporosis treatment, the roles of light therapy and melatonin supplementation have gained increasing attention (Qian et al., 2025). The SCN functions as the master pacemaker of circadian rhythms and is sensitive to photic cues (Song et al., 2018b; Lucassen et al., 2016; Yuan et al., 2026). Light represents the most important environmental cue for endogenous circadian entrainment (Qian et al., 2025; Yuan et al., 2026). On one hand, ultraviolet (UV) radiation from sunlight catalyzes the conversion of 7-dehydrocholesterol in the skin to bioactive 1,25-dihydroxyvitamin D_3_ (1,25-(OH)_2_D_3_), which regulates osteoblast differentiation (Bikle, 2025; Carlberg et al., 2023). On the other hand, clinical and animal studies indicate that UV exposure can increase serum 25-hydroxyvitamin D [25(OH)D] levels in osteoporotic patients or mice, thereby correcting skeletal metabolic imbalance (Makida et al., 2020; Ochiai et al., 2021). Importantly, melatonin, a key SCN-regulated hormone, is modulated by both circadian clocks and environmental light and can promote bone formation while inhibiting bone resorption (Lu et al., 2025; Qian et al., 2025; Yang et al., 2022; Li et al., 2019). Chronic nighttime light exposure in adults has been shown to disrupt bone metabolic homeostasis and increase osteoporosis risk (Touitou et al., 2017). Consequently, rhythmically timed light exposure and melatonin supplementation can exert therapeutic effects on osteoporosis.

Beyond light therapy and melatonin, rhythmically timed exercise and dietary interventions are also effective in modulating circadian clocks (Qian et al., 2025). Physical activity serves as a potent stimulus for bone formation (Qian et al., 2025). Evidence suggests that the timing of exercise influences its osteogenic effect, with postprandial exercise, particularly downhill exercise at 11:00 or 18:00, enhancing bone anabolism compared to preprandial activity (Qian et al., 2025; Borer et al., 2019). Moreover, high-impact exercises (e.g., jumping, running) and whole-body vibration (WBV) produce significant mechanical loading, which has been demonstrated to improve bone mineral density (BMD) more effectively than low-impact activities (Qian et al., 2025). For instance, postmenopausal women undergoing high-intensity exercise combined with WBV exhibited significantly increased lumbar and femoral neck BMD, whereas low-impact activities such as swimming did not yield significant improvements (Sañudo et al., 2017). Similarly, the timing of food intake influences skeletal metabolism (Qian et al., 2025). Synchronizing feeding windows with metabolic rhythms can modulate the expression of bone-related genes, indirectly improving the balance between bone resorption and formation. Studies have shown that rhythmically timed dietary restriction optimizes systemic energy metabolism and endocrine regulation, further modulating OC circadian function and alleviating osteoporosis symptoms (Qian et al., 2025).

In summary, environmental and lifestyle interventions aimed at reestablishing central–peripheral clock synchrony represent an important complementary strategy for osteoporosis management. Light therapy, melatonin supplementation, rhythmically timed exercise, and dietary regulation can collectively modulate bone metabolism, attenuate hyperactive OC activity, and restore bone homeostasis. These interventions not only complement conventional pharmacotherapy but also provide novel avenues for individualized treatment, promoting comprehensive patient recovery (Table 1).

## 6 Conclusion and perspectives

This review systematically summarizes the structural and functional characteristics of the OC lysosome–resorptive apparatus, as well as the hierarchical regulation exerted by circadian clocks, elucidating their roles in PMOP, senile osteoporosis, and inflammation/glucocorticoid-associated osteoporosis. OC lysosomes achieve precise localization of the bone resorption interface through dynamic regulation of acidic microdomains, while multi-layered rhythmic control from central clocks, peripheral clocks, and cell-autonomous clocks dictates the diurnal pattern of bone resorption. Circadian disruption can lead to lysosomal homeostasis impairment, reduced TFEB activity, and misaligned acidic microdomains, thereby exacerbating OC hyperactivity and bone loss.

Based on these mechanisms, osteoporosis interventions can be approached through multidimensional strategies. Chronopharmacological studies indicate that optimizing the timing of anti-resorptive agents, such as BPs and Denosumab, to align with nocturnal peaks of bone resorption can enhance their inhibitory efficacy on OC activity while also considering gastrointestinal bioavailability. Interventions targeting clock genes, including REV-ERBα agonists SR9009, CRY stabilizers, and BMAL1 modulators, can restore OC-autonomous clocks, thereby suppressing excessive differentiation and abnormal lysosomal remodeling at their origin. Lysosomal homeostasis restoration strategies, such as temporally coordinated activation of TFEB and rhythmic regulation of V-ATPase, can reduce pathological over-degradation of bone matrix while preserving the physiological rhythm of bone resorption. Furthermore, environmental and lifestyle interventions, including light therapy, melatonin supplementation, rhythmically timed exercise, and dietary time management, can reestablish central–peripheral circadian synchrony, indirectly modulating OC lysosomal activity, improving bone metabolic balance, and providing complementary approaches for individualized management.

Looking forward, further elucidation of the molecular coupling between core oscillators and lysosomal function is warranted, along with systematic characterization of circadian features across different osteoporosis subtypes. Clinical translation of chronopharmacology and clock gene-targeted interventions will require high-precision circadian monitoring and personalized dosing strategies, while the temporal “window” for natural compounds and lifestyle interventions also requires validation in clinical and animal models. Integrating circadian regulation with lysosomal function remodeling offers a promising multidimensional strategy to shift osteoporosis management from simple “bone mass maintenance” toward “rhythm-based bone resorption modulation,” enabling mechanism-driven precision therapy and providing both theoretical foundation and practical guidance for future clinical interventions and novel drug development.
